# The Effect of Ongoing Exposure Dynamics in Dose Response Relationships

**DOI:** 10.1371/journal.pcbi.1000399

**Published:** 2009-06-05

**Authors:** Josep M. Pujol, Joseph E. Eisenberg, Charles N. Haas, James S. Koopman

**Affiliations:** 1 Department of Epidemiology, University of Michigan, Ann Arbor, Michigan, United States of America; 2 Center for the Study of Complex Systems, University of Michigan, Ann Arbor, Michigan, United States of America; 3 Department of Civil, Architectural and Environmental Engineering, Drexel University, Philadelphia, Pennsylvania, United States of America; Emory University, United States of America

## Abstract

Characterizing infectivity as a function of pathogen dose is integral to microbial risk assessment. Dose-response experiments usually administer doses to subjects at one time. Phenomenological models of the resulting data, such as the exponential and the Beta-Poisson models, ignore dose timing and assume independent risks from each pathogen. Real world exposure to pathogens, however, is a sequence of discrete events where concurrent or prior pathogen arrival affects the capacity of immune effectors to engage and kill newly arriving pathogens. We model immune effector and pathogen interactions during the period before infection becomes established in order to capture the dynamics generating dose timing effects. Model analysis reveals an inverse relationship between the time over which exposures accumulate and the risk of infection. Data from one time dose experiments will thus overestimate per pathogen infection risks of real world exposures. For instance, fitting our model to one time dosing data reveals a risk of 0.66 from 313 *Cryptosporidium parvum* pathogens. When the temporal exposure window is increased 100-fold using the same parameters fitted by our model to the one time dose data, the risk of infection is reduced to 0.09. Confirmation of this risk prediction requires data from experiments administering doses with different timings. Our model demonstrates that dose timing could markedly alter the risks generated by airborne versus fomite transmitted pathogens.

## Author Summary

We model the relationship between the temporal patterns of pathogen exposure and infection take off within people. Since different routes of transmission (e.g., airborne versus surface transfer routes) may result in different temporal patterns of exposure, this model helps to better compare the risks of transmission from one person to another through these different routes. Previous models assumed that the risk of infection is the same whether pathogens are inoculated all at once or over one day. Our model, in contrast, captures how one pathogen affects the potential of immunity to keep concurrently or subsequently arriving particles from initiating an infection. Since the pattern of timing of airborne and surface spread pathogen arrivals differ, our model shows that each airborne pathogen could carry less risk than each surface transmitted pathogen. Unfortunately, data to fully fit our model are not currently available. Therefore new experiments will have to be conducted where doses are given across different temporal windows.

## Introduction

Microbial risk assessment models are valuable tools for estimating the risks associated with exposures to pathogens in the environment pathogens [1]. Central to this estimate is a dose-response model that predicts the probability of infection given a dose exposure magnitude. In current microbial risk assessment models dose accumulates over time and the probability of infection is based on the total accumulated dose over that period of time [2]–[4]. This assumes that each pathogen particle carries a risk of infection that is independent of when other pathogens have arrived to a host; i.e., three exposures to dose X generate the same total risk as one exposure to a 3× dose. We put forth an alternative dose response model that assumes the current capacity of immune effectors to control an arriving pathogen should be affected by 1) how many effectors are occupied fighting previously or simultaneously arriving pathogens, 2) how many effectors have been depleted in fighting previously arriving pathogens, and 3) how many effector reinforcements have arrived due to usual effector turnover rates or due to a stimulus from prior pathogen exposure.

If dose-timing effects arise from such immune effector dynamics, then infection-risk calculations that do not take these dose-timing effects into account could lead to errors. For example, errors could arise in models of influenza transmission as follows. Pathogens arriving to a host via aerosols do so more frequently but at lower doses than pathogens arriving via hand or fomite mediated inoculations. Models of influenza transmission that do not account for dose-timing effects, such as the model by Atkinson and Wien [4], might misdirect influenza control resources to masks from hand hygiene. Models that assume independent single dose effects will require more extreme cleaning to reduce risks to acceptable levels than models capturing immune effects on dose timing.

Evaluating the potential importance of such dose-timing effects is difficult for two reasons. First, immune control of pathogens is complex; not enough detailed knowledge regarding that complexity is available to provide a high degree of confidence in a-priori causal model predictions. Second, there is almost no direct observational data documenting the presence or absence of dose-timing effects. Although various studies have given pathogen exposure doses over time [5]–[10], only Brachman et al. [11], has been conducted in a manner that allows one to calculate risks for comparable doses administered over different temporal windows.

In this paper we have taken an approach intended to stimulate science that will address both of these issues. We develop a simple model that illustrates the need to generate new data that can describe dose-timing effects while at the same time providing a base upon which to build more realistic models that incorporate more data and theory on immunity. Our model addresses immune control of pathogens between the time pathogens arrive at a host and the time they are either eliminated or have multiplied enough so that an acquired immune response will be needed for control.

We make our model general enough to capture dynamics of pathogen control that might arise from established antibodies and T-cells, macrophages, polymorphonuclear leukocytes, plasma cells, dendritic cells, complement cascades, chemokines, interleukins, interferons, toll like receptors, and other diverse elements affecting immunity. But we lump all these mediators of pathogen control into a highly abstract entity we label as immune effectors. We assume that the dynamic effects of limited immune effector numbers are similar whether the limitation arises from immune effectors being occupied with previously arrived pathogens or from prior consumption of immune effectors in their process of killing pathogens. Therefore we only model the latter source of immune effector limitations. The resulting model is one where any single pathogen always has some chance of initiating an infection but the risk of infection associated with each additional pathogen exposure can markedly increase at higher pathogen doses given over short temporal windows. The exact dynamics of our model will vary as realistic details are added to it. Our goal here is simply to illustrate the importance and inevitability of immune mediated dose-timing effects so as to stimulate further empirical and theoretical work.

The structure of the paper is as follows: in the methods section we describe the Cumulative Dose model and analyze its dynamics. In the results section we use the Cumulative Dose model to fit experimental data assuming a fixed temporal exposure window to simulate the archetypical single dose experiment of dose-response trials. Using the estimated model we show the effect of changing the length of the temporal exposure window. Finally, the conclusions and future research are presented in the discussion section.

## Methods

### Cumulative Dose Model

The model is based on a stochastic population of individual pathogens and immune effectors. Since the focus of our analysis is how small populations of pathogens either die out or lead to infection initiation, we cannot rely on the mean-field solution provided by the deterministic framework [12]–[14].

The state of the system is defined by the pair (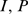) representing the number of immune effectors and the number of pathogens, in any single host, respectively. The system is defined by the following set of state transitions:
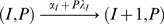
(1)

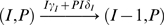
(2)

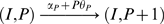
(3)

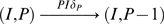
(4)


The number of immune effectors 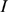 can increase at: 1) a rate 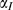, which models the constant arrival of immune effectors regardless of the current state of the immunological system; and 2) a rate 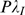, which models the recruitment of immune effectors in the presence of pathogens. This term is intended to reflect cytokine induced recruitment of remote immune effectors to a pathogen invasion site and not acquired immunity. We assume that the relative endpoints of infection takeoff or pathogen elimination are reached before an acquired immune effect comes into play. Immune effectors decrease either at a natural death rate 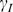, or at a mass-action deactivation rate due to the encounter with pathogens 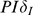.

The number of pathogens 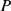 can increase by reproduction at a rate 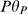 or by arrival during the inoculation period at a rate 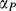. Here 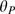 represents the net reproduction rate that aggregates birth and death rates. Pathogen numbers decrease due to interaction with immune effectors as a mass-action deactivation process at the rate 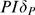.

### Dynamics of the Cumulative Dose Model

The initial state of the system is set to 
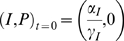
. No chronic low-level exposures or remaining pathogens from prior exposures are considered. The system starts from the clean state: no pathogens and the stationary number of immune effectors in the absence of pathogens. The inoculation process is characterized by the dose of exposure 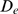 and the temporal exposure length 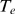; i.e., the dose that is composed by 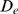 pathogens is inoculated into the host during a period of 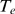 time units. Therefore, the arrival of external pathogens is modeled as the rate 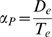 during the inoculation period. Once inoculation has finished the pathogen arrival rate becomes zero. Thus, the rate 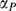 depends on time and is defined as
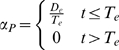



During 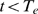, the pathogens, 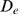 arrive over a continuous time in the presence of the immunological response to those pathogens. Once the inoculation has finished, only the immunological response remains. We set the unit of time to an hour. That keeps us in the range where we think exposure fluctuations are making a difference and out of the range where adaptive immune system feedbacks come into play.

Due to stochastic effects and the fate of a relatively small population of pathogens and immune effectors, the same inoculation dose 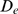 administered in the same time frame 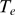 does not necessarily have the same outcome. Each replication (i.e. run) of the model corresponds to a dose trial on a new subject. All the numerical results are the average of 10^4^ runs of the Cumulative Dose model implemented with the Gillespie algorithm [15] using *C*. The criteria to stop the simulation is either extinction of pathogens after the inoculation period (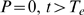) or pathogens diverging to a very large number, 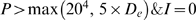, corresponding to no infection and infection respectively. The probability of infection for a pair 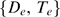 is the proportion of simulations that diverge to a large number as opposed to equilibrating to the state of no pathogens.

**Figure 1:**
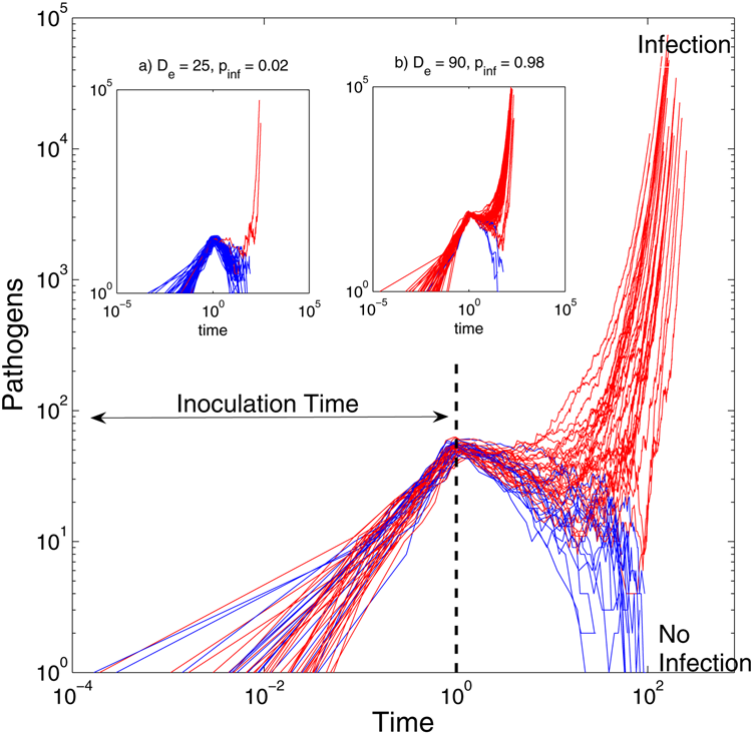
Evolution of the number of pathogens over time for a characteristic parameter set 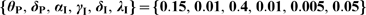. Each line represents an individual replicate with the same parameter set (100 in total). The fraction of replicates in which the number of pathogens diverge towards infinity, as opposed to going extinct, is equivalent to the probability of infection (*p_inf_*) for the dose 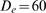 (main graph, 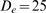 and 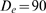 for the insets a) and b) respectively). Temporal exposure length is fixed at *T_e_* = 1 hour. Probability of infection is 0.67, 0.02 and 0.98 for the main graph, the inset a), and the inset b) respectively.

Figure 1 illustrates the stochastic process effects on pathogen dynamics given a fixed time of exposure for different inoculation doses. The main plot in this figure is the time course of the number of pathogens for 100 independent dose trials given a dose of 60 pathogens administered over one unit of time. The number of pathogens steadily grow during the inoculation period, from 0 to 1, since the rate of arrival of pathogens (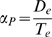) is much faster than immunological killing of pathogens. Once the entire dose has been inoculated at time = 1, the external arrival of pathogens stop (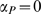) and the immunological response dominates the rest of the dynamics. In this particular case, the population of pathogens becomes extinct in 33 cases out of 100, thus, the probability of infection given a dose of 60 pathogens over 1 unit of time is 0.67. Analogously, for a dose of 25 the probability of infection is 0.02 and for a dose of 90 the probability of infection is 0.98 (insets of Figure 1).

### Temporal Exposure Length

Figure 1 illustrates how the Cumulative Dose model yields higher probability of infection when the inoculated dose is increased. The length of time over which the dose is administered, 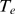, also plays a crucial role in the probability of infection. At one extreme where all the pathogens were inoculated at once (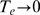), the immune system has no time to react, and the initial state of the system is 
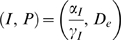
. From this initial state, the immunological response dynamics determines the fate of the pathogens: either extinction or unbounded growth of pathogens diverging towards infinity.

**Figure 2:**
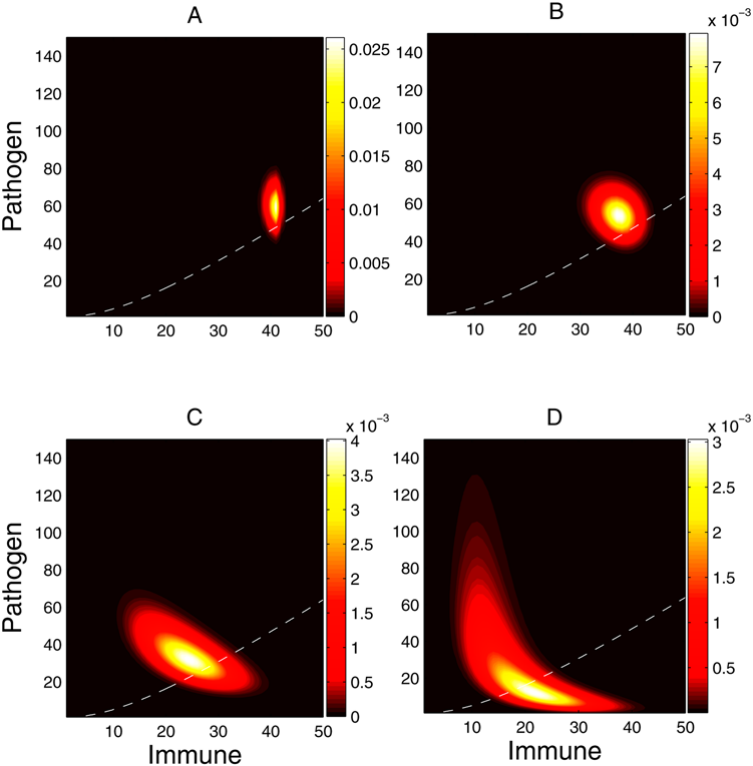
State probability distribution at the end of inoculation (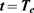) for a dose of 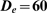 and temporal exposure length of 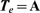) 0.1 h, B) 1.0 h, C) 10.0 h and D) 50.0 h. The distribution of probabilities if 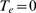 would be 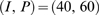 given the parameters of the system are 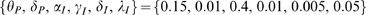. The dashed white line is the separatrix of the deterministic version of the model (see subsection Deterministic Analysis); if the system were deterministic once inoculation has been completed, the states that fall below the separatrix would end up in no infection, and the states above would end up in infection.

For 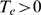, however, the initial state after all pathogens have been inoculated (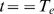) is not the expected 
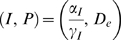
, but rather a distribution of probabilities over the space of possible states. Figure 2 shows the stochastically determined distribution of system states at the point in Figure 1 where the exposure time has just ended. It illustrates the effect of different temporal exposure lengths, ranging from 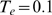 (six minutes) to *T_e_* = 50 hours. Panel B shows this point of time for the settings in Figure 1. The longer the exposure length, the larger will be the variance in the distribution of probabilities. Furthermore, a longer exposure length also affects the average state after inoculation. Both the pathogen levels and the immune effector levels decrease from the instantaneous inoculation values as the exposure window length increases. But the balance between these increasingly favors the immune effectors. Longer temporal exposure lengths dilute the arrival rate of external pathogens, 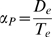. Consequently the immunological response has more time to neutralize the existing pathogens before the arrival of new pathogens. On the other hand, as the temporal exposure lengths decrease, an increased number of immune effectors are consumed in killing pathogens, leading to a higher probability of unbounded growth of pathogens, and thus infection.

For 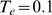 and 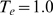 the average state after inoculation is very close to the ideal instantaneous inoculation, 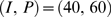. To better understand the dynamics once inoculation is over, we included the numerically calculated separatrix as if the system were deterministic (red-dashed line in Figure 2). Although this separatrix is only truly valid for the analogous deterministic model, it indicates the probable fate of different initial states. For the deterministic system, the separatrix separates those states that go to infection from those that do not (see subsection on Deterministic Analysis). As temporal exposure length increases, the distribution of probabilities gravitates towards the space of states that go to no-infection (below the separatrix).

### Deterministic Analysis

Further understanding of the stochastic dynamics of the Cumulative Dose model can come from a deterministic description of the system that assumes a continuous large number of immune effectors and pathogens. We focus our analysis on the dynamics after the inoculation period, so 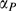 is set to 0 and removed from the equations. This analysis on the deterministic version helps illustrate the interactions between pathogens and immune effectors that result either in infection or extinction of pathogens.

The stochastic system is fully described by a multivariate master equation [16], which can be expanded in a deterministic formulation known as *macroscopic law*. The deterministic version of the cumulative dose model is as follows,
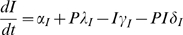
(5)

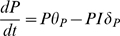
(6)where 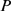 and 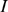 are continuous variables of the population of pathogens and immune effectors respectively. The fixed points of the deterministic version of the cumulative dose model are 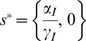
where the pathogen has been eliminated and immune effectors are in equilibrium and 
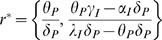
 where the forces of pathogen growth are balanced by immune dynamics affecting pathogen death. Note that in the stochastic analyses of this model as in Figure 1, this point is never reached. Instead simulations are terminated when growth takes off toward this point. A simple analysis of the stability of the fixed points reveals the space of parameters in which the solution is well-defined.

The point 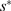 is the equilibrium of no infection—the equilibrium of the system in the absence of pathogens. When the system gravitates towards 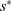 the immunological system prevents pathogens from growing, resulting in pathogen extinction and therefore no infection.

To evaluate the stability of the fixed point, we formulate the Jacobian matrix of the system of equations on 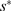.
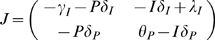
(7)


For a stable equilibrium, both Eigenvalues of the Jacobian matrix need to be negative, or equivalently, the matrix must have a negative trace and a positive determinant. For the trace of the Jacobian to be negative the condition 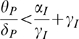 must be true. Since the positive determinant condition, 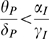, is more restrictive it subsumes the condition for a negative trace.

The second fixed point 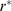 is only well-defined when both 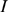 and 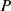 are positive, since negative number of pathogens and immune effectors are impossible. The number of pathogens is only positive when 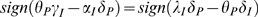. Given the condition of a positive determinant, 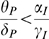, the sign can only be negative, consequently 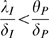. Therefore, the system is well defined — i.e. has a stable equilibrium at no infection and with both fixed points in the positive quadrant — only when the following condition 8 is met
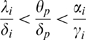
(8)


**Figure 3:**
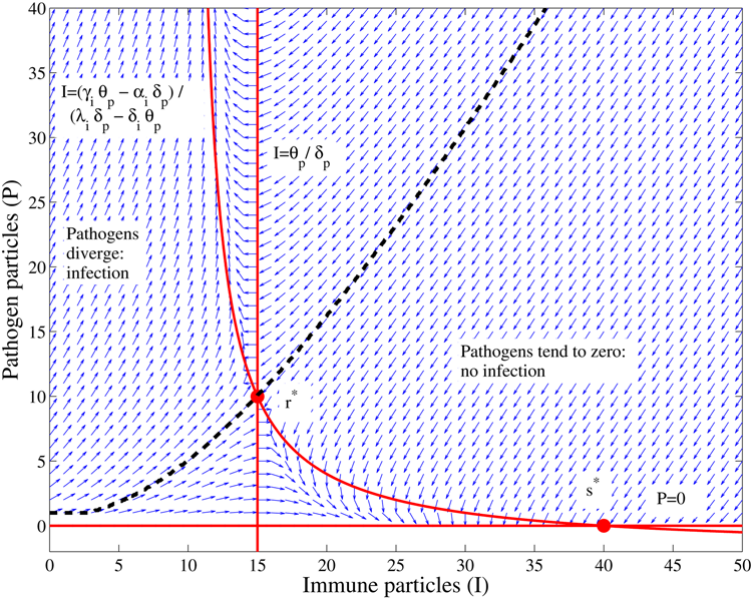
Vector field plot of the deterministic cumulative dose model for a characteristic parameter set 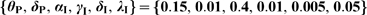. To avoid overlaps of the vectors they have been normalized. The solid red lines are the nullclines, the intersections of the nullclines are the fixed points 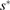 (stable pathogen elimination equilibrium) and 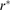 (unstable saddle point equilibrium). The dash black line is the separatrix that separates those configurations that will go to non-infection equilibrium, 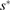, and those that will diverge in the number of pathogens resulting on infection. The separatrix has been calculated numerically.

Once we determine the stability of 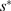 we need to characterize the second fixed point 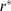. After some basic algebra, the determinant of the Jacobian matrix for 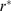 can be expressed as follows: 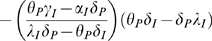. Given condition 8, both terms are positive, which makes the determinant negative. As a result the Eigenvalues of the Jacobian are real with different signs. Therefore, 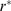 is a saddle point as shown in Figure 3.

The vector field in Figure 3 illustrates the dynamics of the cumulative dose after the inoculation period. The probability of being in a given state after inoculation is shown in Figure 2. If the system were deterministic then we could anticipate the probability of infection by summing the probability of those states below the separatrix. This does not hold for the stochastic Cumulative Dose model. Nonetheless, the deterministic vector field, shown in Figure 3, serves as an approximate description of what happens in the stochastic model.

For instance, let us take the probability distribution of states when centered at 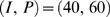, i.e., 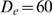 and 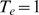. The typical dynamic results in the decrease in number of pathogens and immune effectors, gravitating towards the saddle point 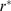, from which it will bifurcate to the stable point of no-infection 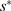, or an unbounded growth of pathogens. In the case of 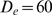 and 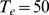, most of the states are already very low in pathogens, and consequently the number of immune effectors will eradicate the few pathogens still existing and go to the stable equilibrium of no infection. However, there is a non-zero probability, albeit small, of being in a state with a large number of pathogens and a small number of immune effectors. In this case, stochastic perturbations aside, the pathogens will keep multiplying producing infection in the host.

## Results

### Analysis of Exposure Dose Risks

In this section, we fit empirical data on multiple pathogens for the single event inoculation scenario. Next, we extend our analysis to incorporate different temporal exposure windows and patterns of inoculation.

#### Fitting experimental dose-response data

We selected three different pathogen datasets: 1) poliovirus [17], 2) *Cryptosporidium parvum* [18] and 3) rotavirus [19]. Analyses of these three datasets are found elsewhere [20].

Several statistical models based on the empirical data have been proposed to describe dose-response data. The most common models are the Exponential model [1]:
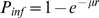
(9)where *μ* is the inoculation dose and *r* is the per pathogen risk, and the Beta-Poisson model [21]:
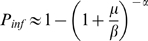
(10)where *μ* is the inoculation dose and *α* and *β* are parameters of the beta distribution that describes the host pathogen interaction. Other models such as Log-Logistic and Weibull have been used, but not as commonly.

For parameter estimation we used a classical genetic algorithm [22]. The fitness function of the genetic algorithm was the mean square error (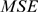). We fixed the exposure time (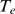) of our inoculated dose (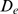) to 1.0 time units in order to emulate the empirical dose-response experiments in which the dose is inoculated in a single shot; i.e., a very short exposure. We present the best fitting curves and discuss their limitations in the subsection “The Effect of Temporal Exposure Length”. Then, given these best fitting parameter values, we present the effect of different temporal exposure windows on the final probability of infection.

### Poliovirus

**Figure 4:**
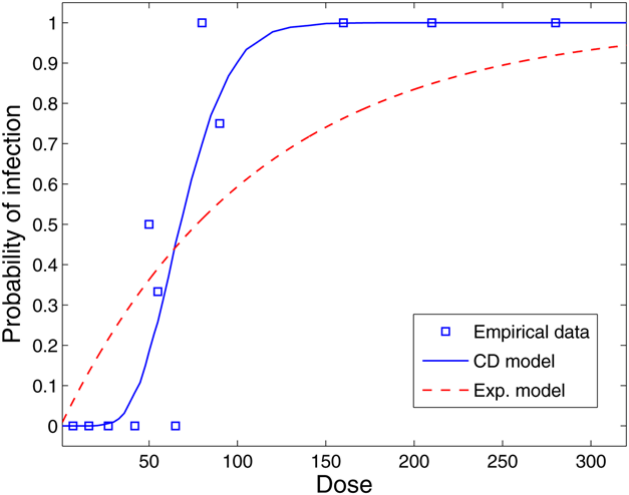
Dose-response curves based on the Exponential Model (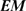) and the Cumulative Dose model (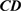) compared to the experimental dataset for Poliovirus type 1 (squares). The estimated parameters are 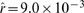 for the Exponential model [3] and 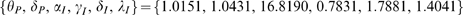 for the Cumulative Dose model.

The first empirical dataset to which we apply the Cumulative Dose model is Poliovirus type 1 [17]. The cohort for this experiment was 32 2-month-old infants. Inoculation was oral. Figure 4 and Table 1 show the fit alongside a fit to the Exponential model (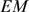) according to [18].

**Table 1:** Probability of infection from experimental data for Polivirus type 1 (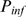) compared to the probability of infection based on the Exponential model (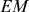) and the Cumulative Dose model (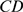).

Dose	No. of subjects	No. Infected	Fraction Infected 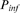	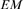 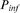	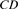 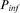
**7.0**	1	0	0.0	0.0617	0.0
**16.0**	2	0	0.0	0.1355	0.0
**27.0**	2	0	0.0	0.2178	0.0062
**42.0**	1	0	0.0	0.3176	0.0831
**50.0**	6	3	0.50	0.3656	0.1840
**55.0**	3	1	0.333	0.3938	0.2582
**65.0**	6	0	0.0	0.4465	0.4523
**80.0**	1	1	1.0	0.5171	0.6992
**90.0**	4	3	0.75	0.5591	0.8189
**160.0**	3	3	1.0	0.7668	0.999
**210.0**	2	2	1.0	0.8521	1.0
**280.0**	1	1	1.0	0.9218	1.0

The estimated parameters are 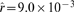 for the Exponential model [3] and 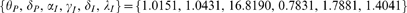 for the Cumulative Dose model.

### Cryptosporidium

**Figure 5:**
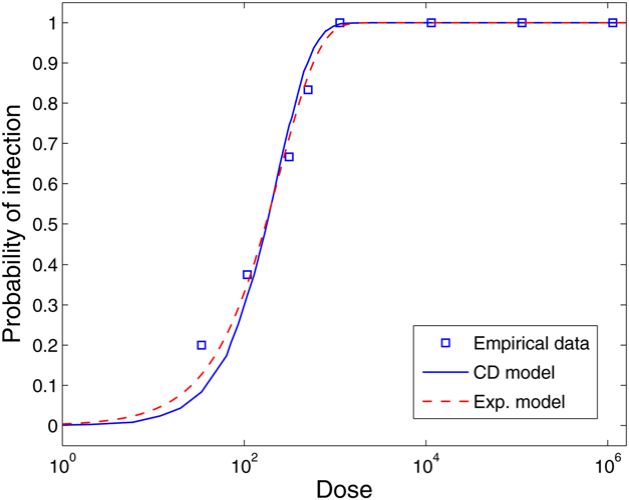
Dose-response curves based on the Exponential Model (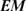) and the Cumulative Dose model (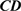) compared to the experimental dataset for *Cryptosporidium parvum* (squares). The estimated parameters are 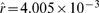 for the Exponential model [30] and 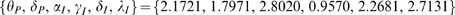 for the Cumulative Dose model.

The cohort for the *Cryptosporidium parvum* study [18] was 35 healthy subjects (12 men and 17 women, age range between 20 and 45 years). The strain was an isolate from a calf and the inoculums were orally administered via capsules. Figure 5 and Table 2 show the fit alongside a fit to the Exponential model (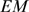) according to [20].

**Table 2:** Probability of infection from experimental data for *Cryptosporidium parvum* (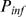) compared to the probability of infection predicted by the Exponential model (EM) and the Cumulative Dose (CD) model.

Dose	No. of subjects	No. Infected	Fraction Infected 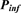	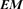 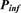	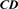 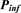
34	5	1	0.2	0.1273	0.0848
108	8	3	0.375	0.3511	0.3173
313	3	2	0.6667	0.7145	0.7421
504	6	5	0.8333	0.8671	0.9065
1129	2	2	1.0	0.9891	0.9972
11460	3	3	1.0	1.0	1.0
113900	1	1	1.0	1.0	1.0
1139000	1	1	1.0	1.0	1.0

The estimated parameters are 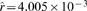 for the Exponential model [30] and 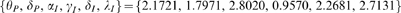 for the Cumulative Dose model.

### Rotavirus

Finally, we tested the Cumulative Dose model against a dataset for Rotavirus [19].

The cohort for rotavirus was 62 adult males, 18 to 45 years old. The inoculation was oral. Unlike the previous dose-response empirical datasets, neither the Cumulative Dose model nor the Exponential model produce a good fit. The Beta-Poisson model (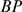) was statistically a better fit than the Exponential model [20].

**Figure 6:**
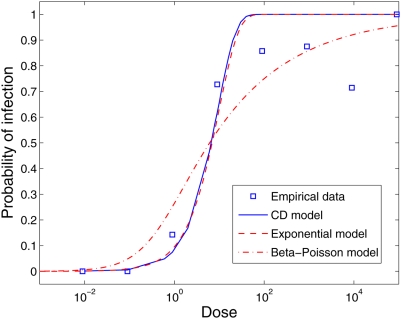
Dose-response curves based on the Exponential Model (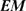), the Beta-Poisson model (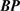) and the Cumulative Dose model (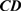) compared to the experimental dataset for Rotavirus (squares). The estimated parameters are 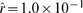 for the Exponential model, 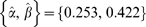 for the Beta-Poisson model [31] and 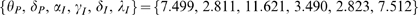 for the Cumulative Dose model.

Both the Exponential and the Cumulative Dose model increase too rapidly in relation to the probability of infection of 1; i.e. these models cannot maintain a non-zero or non-one probability of infection for a dose range of several orders of magnitude. Conversely, the Beta-Poisson model does not suffer from this limitation since its convergence to 1 is slower, providing a wider range of variance (Figure 6 and Table 3).

**Table 3:** Probability of infection from experimental data for Rotavirus (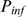) compared to the the Exponential, Beta-Poisson and Cumulative Dose models.

Dose	No. of subjects	No. Infected	Fraction Infected 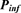	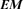 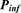	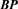 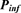	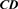 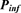
9×10^−3^	5	0	0.0	0.0009	0.0053	<0.001 (*)
9×10^−2^	7	0	0.0	0.009	0.0477	0.0053
9×10^−1^	7	1	0.1428	0.0861	0.2509	0.0740
9	11	8	0.7273	0.5934	0.5442	0.6175
9×10^1^	7	6	0.8571	0.9999	0.7428	0.9999
9×10^2^	8	7	0.875	1.0	0.8562	1.0
9×10^3^	7	5	0.7143	1.0	0.9197	1.0
9×10^4^	3	3	1.0	1.0	0.9551	1.0

The estimated parameters are 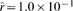 for the Exponential model, 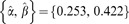 for the Beta-Poisson model [31] and 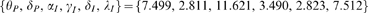 for the Cumulative Dose model. (*) The dose in the original trial was administered in concentrations, to work with discrete pathogens as required by the Cumulative Dose model, we assumed that the concentration of 9×10^−2^ is equivalent to 9 pathogens. As a consequence the concentration of 9×10^−3^ could not be tested since it is a fraction of a pathogen. The probability of infection for a single pathogen is 10^−3^. This assumption is only required by the Cumulative Dose model.

A possible explanation of the poor fit of the Cumulative Dose model is the high degree of acquired immunity to Rotavirus and the changing serotype profile circulating within populations [23]. Unlike the polio virus study, the rotavirus cohort consisting of adults (18–45 years old), is likely to have been exposed multiple times to various rotavirus serotypes [24]. Such heterogeneity in susceptibility flattens out dose response curves beyond what can be captured by exponential dose response models or this Cumulative Dose response model.

### The Effect of Temporal Exposure Length

In the previous subsections we fixed temporal exposure length, 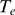, to 1 hour, and assume that this is the time corresponding to the single shot inoculation, analogous to existing experimental dose-response trials. In this section, we present simulations for a range of different temporal exposure lengths, illustrating how longer times affect the dose response curve. The model is set to the parameters that provided an optimal fit for a temporal exposure length of 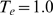.

**Figure 7:**
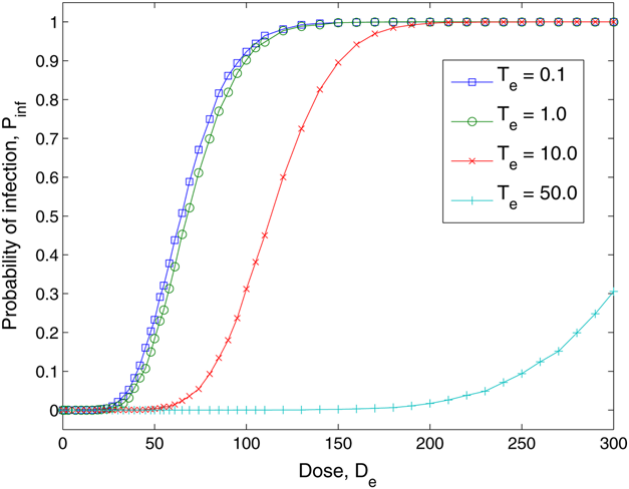
Predicted effects of varying exposure times (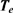) when inoculated with Poliovirus type 1. Parameters are defined as stated in Figure 4.

Figure 7 shows the dose-response curves for Poliovirus type 1 for different lengths of exposure for the estimated parameters used in Figure 4 to fit the experimental data for the condition T_e_ = 1.0: 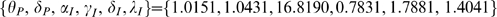. As the exposure length increases, the probability of infection decreases dramatically. Therefore, assuming that the unit of time is one hour, and this is the equivalent for a dose that is administered in a single shot, the probability of infection generated by the Cumulative Dose model for a dose of 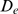 of 90 pathogens administered in one hour is 0.82. If the dose were administered not in one hour, but uniformly over ten hours the probability of infection would be 0.18. If the dose were administered over fifty hours the probability of infection would be reduced to 0.0001. To obtain the same probability of infection for a ten hours inoculation period instead of one, we would require a dose of 139 pathogens instead of 90.

**Figure 8:**
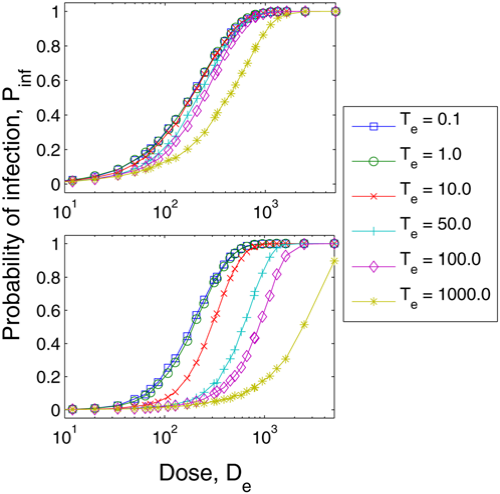
Predicted effects of varying exposure times (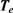) when inoculated with *Cryptosporidium parvum*. The top graph comes from simulations using the parameter set defined in Figure 5. The bottom graph comes simulations using the parameter set 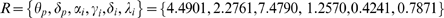.

Because data on the impact of temporal patterns of inoculation are currently not available, a model with dose-time dependence such as ours is not identifiable [25]; i.e., the model can be fit to existing single dose empirical data with many different parameters sets. For example, in Figure 8 we show model simulation results for *Cryptosporidium parvum* for two different parameter sets. Both parameters sets have a similar fit to the *Cryptosporidium parvum* dataset when 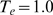 (mean square error using 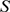 and 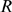 is 3.5×10^−3^ and 9.7×10^−3^ respectively). For values of 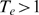, however, the dose response relationships of the two parameter sets diverge. Parameter set 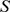 is much less sensitive to exposure time than 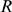 due its slower dynamics. Using parameter set R, pathogens proliferate faster, are being eliminated by each immune effector more quickly, are recruiting fewer immune effectors, and are eliminating immune effectors at a slower rate. On the other hand, using parameter set R, the natural rate of turnover of immune effectors is more rapid. We cannot argue at this point which is the most plausible configuration since identifiability cannot be resolved without data from dosing trials for different exposure lengths.

### The Effect of Dosing Patterns over the Exposure Window

In this section we relax the assumption that pathogens are inoculated at a fixed rate. We allow variation both in dose magnitude and length of exposure time, in order to capture a more realistic exposure scenario.

The temporal pattern of inoculation of pathogens within a host depends both on the behavior of the host and the contamination of the environment the host interacts with. For instance, a susceptible host in a venue contaminated with influenza will be exposed to pathogens from air and fomites. However, the temporal patterns of exposure for these two modes of transmission are different. The host is likely to receive a small dose with every breath when breathing contaminated air. In fomite mediated transmission, however, the touching of a mucous membrane with contaminated fingers, for example, is likely to transmit a larger but less frequent dose.

To illustrate this effect we devised an experiment where both the total inoculated dose 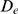 and the exposure time length 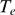 are fixed. The only parameter that varies is the number of inoculation events, 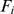, which ranges from 1 to the total dose 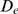. Consequently, once the number of inoculations events is determined, the dose inoculated in each event is 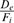 and the rate at which inoculation occur is 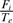.

**Figure 9:**
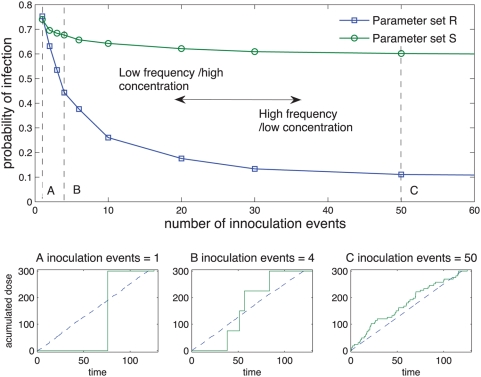
Predicted effects of different temporal patterns of exposure when inoculated with *Cryptosporidium parvum*. The main figure displays the probability of infection as function of the number of inoculation events. The line with circular markers comes from simulation results using the parameter set defined in Figure 5, and the line with square markers comes from simulation results using the parameter set 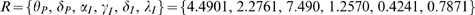 The insets below demonstrate three temporal patterns for three different patterns of inoculation events: A = 1, B = 4 and C = 50 events respectively. The solid line represents one instance of the 5000 replicas used in the experiment. The dashed line represents the average of dose inoculated over time.

Figure 9 shows the results of this experiment where the same parameter sets are used as in Figure 8. The pathogen is *Cryptosporidium parvum*, and the same two different parameters sets, *S* and *R*, are used to inform the cumulative dose model. The total dose inoculated is set to 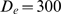 and the temporal exposure length is set to *T_e_* = 120.0 hours.

For both parameter sets S and R we observe the same behavior: infectivity decreases as the frequency or number of inoculations events increases. The temporal pattern more likely to be associated with fomite transmission (low frequency and high dose, Figure 9.B) is more likely to produce infection than the patterns associated with airborne transmission (high-frequency and low dose, Figure 9.C) .

For parameter set *R*, the probability of infection if the dose is inoculated with a single exposure (Figure 9.A) is 0.752. The same dose inoculated over 4 events, where each event is one fourth of the total dose (Figure 9.B), reduces the probability of infection to 0.443. In addition, if the dose is inoculated over 50 events (Figure 9.C) the probability decreases to 0.111. For parameter set *S*, the reduction of the infection probability is less pronounced: 0.740, 0.676 and 0.601 for 1, 4 and 50 inoculation events respectively.

In previous sections we showed that longer temporal exposure lengths decrease infectivity due to the action of the immune system. In this section, we show that not only the duration of the exposure matters, but also the way in which pathogens arrive within that interval can decrease infectivity. These results suggest that risk assessments based on current dose-response data might be over-estimating risk of infection. An important corollary is that risk of infection for a given exposure dose may depend on the route of transmission based on their differences in the pattern of exposure.

## Discussion

We examined a dynamic mechanistic model where immune system effects generated dose response dependence on the timing of doses. The specific aspects of our model that generate these dose-timing effects are: 1) decreases in available immune effectors because they are being eliminated as they kill pathogens; and 2) increases in available immune effectors due to both pathogen dependent and independent recruitment. An additional mechanism resulting in decreases in available immune effectors that is not included in our model could be the time of immune effector engagement with pathogens in the killing process. The dose-timing effects we illustrate would be absent in a model where some effector like a T-cell instantaneously kills pathogens or pathogen generating cells, where no killing capacity is lost with each kill, and where effector dynamics are not otherwise altered by encounters with pathogens. Any such model, however, is highly unrealistic, and therefore we conclude that the dose-timing effects presented in our model could be important and warrant further study.

Dose-timing effects have implications for microbial risk assessment, for infection transmission system modeling, and for the evolution of emerging pathogens. Considering a microbial risk assessment example, the implications of our findings suggest that exposure routes with different dose-timing dynamics could have different risks and therefore result in different clean up protocols for contamination events such as a norovirus outbreak or a Katrina-like disaster. Dose timing could, therefore, affect decisions on which venues to close or what the total dose that workers would be permitted to accrue during a cleanup operation.

Considering modeling infection transmission, the standard approach is to define a contact and a transmission probability per contact while the physical route of transmission is ignored. Modeling the physical route of transmission is important when it is necessary to specify how much transmission is taking place in particular public venues and when specifying which control actions in these venues will reduce transmission. When different routes have different temporal exposure patterns, we demonstrate here that there is considerable potential for immune system effects to alter the ratio by which airborne transmitted and hand-fomite transmitted pathogens generate new infections. If we had data on infection risks under different dose-timing patterns, we could say more precisely how much difference in risk there might be from an airborne and a hand-fomite mediated pathogen. Unfortunately such data is lacking.

The evolution of emerging infection implications derive from the route of transmission effects just discussed. When pathogens first jump species, they are likely to encounter strong innate immune responses to which they must evolve some escape strategy. That means very high transmission doses will be required to sustain transmission and that low dose exposure over longer times such as occurs with airborne transmission will be the most unlikely to be effective in transmitting infection. But, as escape from innate immune responses evolves, the balance could begin to favor airborne transmission which might be more effective in disseminating infection.

We do not have enough dose timing data for any infection to evaluate either the microbial risk assessment implications, the infection transmission system implications, or the emerging infection evolution implications. Any data providing indications of the magnitude of dose-timing effects generated by any type of immunity to any agent would provide an important first step that would at least indicate what range of effects might be expected. Animal studies could compare the risks associated with a single instantaneously delivered dose with the same dose magnitude delivered over extended periods of time. Measurements of specific immune effector dynamics, such as interferon gamma [26] would improve our mechanistic understanding of a cumulative dose effect and indicate how to refine our models for different animal/pathogen systems.

The issue of dose-response trial design is crucial for advancing both quantitative microbial risk assessment and analysis of population infection transmission systems. Due to the absence of a prior theoretical framework, there has been no motivation to conduct dosing trials that take multiple doses and multiple dosing times into account. Now that the potential effects of dose timing have been demonstrated and the practical significance of such measurements for microbial risk assessment and transmission system analyses is more evident, we hope to see such experiments.

## References

[1] Haas CN, Rose JB, Gerba CP (1999). Quantitative Microbial Risk Assessment.

[2] Noakes CJ, Beggs CB, Sleigh PA, Kerr KG (2006). Modelling the transmission of airborne infection in enclosed
spaces.. Epidemiology and Infection.

[3] Eisenberg JNS, Lei X, Hubbard AH, Brookhart MA, Colford JM (2005). The role of disease transmission and conferred immunity in
outbreaks: Analysis of the 1993 Cryptosporidium outbreak in Milwaukee.. American Journal of Epidemiology.

[4] Atkinson MP, Wein LM (2008). Quantifying the routes of transmission for pandemic influenza.. Bull Math Biol.

[5] Ellenberger D (2006). HIV-1 DNA/MVA vaccination reduces the per exposure probability of
infection during repeated mucosal SHIV challenges.. Virology.

[6] Garcia-Lerma JG (2008). Prevention of rectal SHIV transmission in macaques by daily or
intermittent prophylaxis with emtricitabine and tenofovir.. PLoS Med.

[7] Tuckwell HC, Shipman PD, Perelson AS (2008). The probability of HIV infection in a new host and its reduction
with microbicides.. Math Biosci.

[8] Van Rompay KK, Kearney BP, Sexton JJ, Colon R, Lawson JR, Blackwood EJ, Lee WA, Bischofberger N, Marthas ML (2006). Evaluation of oral tenofovir disoproxil fumarate and topical
tenofovir GS-7340 to protect infant macaques against repeated oral
challenges with virulent simian immunodeficiency virus.. J Acquir Immune Defic Syndr.

[9] Van Rompay KK (2005). Attenuated poxvirus-based simian immunodeficiency virus (SIV)
vaccines given in infancy partially protect infant and juvenile macaques
against repeated oral challenge with virulent SIV.. J Acquir Immune Defic Syndr.

[10] Wilson NA (2006). Vaccine-induced cellular immune responses reduce plasma viral
concentrations after repeated low-dose challenge with pathogenic simian
immunodeficiency virus SIVmac239.. J Virol.

[11] Brachman PS, Kaufman AF, Dalldorf FG (1966). Industrial inhalation Anthrax.. Bacteriol Rev.

[12] Rand DA, Wilson HB (1991). Chaotic stochasticity: a ubiquitous source of unpredictability in
epidemics.. Proc Royal Society B.

[13] McKane AJ, Newman TJ (2005). Predator-prey cycles from resonant amplification of demographic
stochasticity.. Physical Review Letters.

[14] Alonso D, McKane AJ, Pascual M (2006). Stochastic amplification in epidemics.. J R Soc Interface.

[15] Gillespie DT (1976). A general method for numerically simulating the stochastic time
evolution of coupled chemical reactions.. Journal of Computational Physics.

[16] van Kampen NG (1992). Stochastic processes in physics and chemistry.

[17] Minor TE, Allen CI, Tsiatis AA, Nelson DB, d'Alesio DJ (1981). Human infective dose determinations for oral poliovirus type 1
vaccine in infants.. Journal of Clinical Microbiology.

[18] DuPont HL, Chappell CL, Sterling CR, Okhuysen PC, Rose JB, Jakubowski W (1995). The infectivity of Cryptosporidium parvum in healthy workers.. The New England Journal of Medicine.

[19] Ward RL, Bernstein DI, Young EC, Sherwood JR, Knowlton DR, Schiff GM (1986). Human Rotavirus studies in volunteers: determination of
infectious dose and serological response to infection.. Journal of Infectious Diseases.

[20] Teunis PFM, van der Heijden OG, van der Giessen JWB, Havelaar AH (1996). The dose-response relation in human volunteers for
gastro-intestinal pathogens.. Rijksinstituut voor Volksgezondheid en Milieu Bilthoven.

[21] Haas CN (1983). Estimation of risk due to low doses of microorganisms: a
comparison of alternative methodologies.. American Journal of Epidemiology.

[22] Holland JH (1975). Adaptation in Natural and Artificial Systems.

[23] Koopman JS, Monto AS (1989). The Tecumseh Study XV: Rotavirus infection and pathogenicity.. American Journal of Epidemiology.

[24] Koopman JS, Monto AS, Longini IM (1989). The Tecumseh Study XVI: Family and community sources of rotavirus
infection.. Am J Epidemiol.

[25] Armitage P, Spicer CC (1956). The detection of variation in host susceptibility in dilution
counting experiments.. Journal of Hygiene.

[26] Howat TJ, Barreca C, O'Hare P, Gog JR, Grenfell TB (2006). Modelling dynamics of the type I interferon response to in vitro
viral infection.. J R Soc Interface.

